# Enhanced p122RhoGAP/DLC-1 Expression Can Be a Cause of Coronary Spasm

**DOI:** 10.1371/journal.pone.0143884

**Published:** 2015-12-01

**Authors:** Takahiko Kinjo, Makoto Tanaka, Tomohiro Osanai, Shuji Shibutani, Ikuyo Narita, Tomohiro Tanno, Kimitaka Nishizaki, Hiroaki Ichikawa, Yoshihiro Kimura, Yuji Ishida, Takashi Yokota, Michiko Shimada, Yoshimi Homma, Hirofumi Tomita, Ken Okumura

**Affiliations:** 1 Department of Cardiology and Nephrology, Hirosaki University Graduate School of Medicine, Hirosaki, Japan; 2 Department of Hypertension and Stroke Medicine, Hirosaki University Graduate School of Medicine, Hirosaki, Japan; 3 Department of Health Promotion, Hirosaki University Graduate School of Health Science, Hirosaki, Japan; 4 Department of Biomolecular Science, Fukushima Medical University School of Medicine, Fukushima, Japan; Niigata University Graduate School of Medical and Dental Sciences, JAPAN

## Abstract

**Background:**

We previously showed that phospholipase C (PLC)-δ1 activity was enhanced by 3-fold in patients with coronary spastic angina (CSA). We also reported that p122Rho GTPase-activating protein/deleted in liver cancer-1 (p122RhoGAP/DLC-1) protein, which was discovered as a PLC-δ1 stimulator, was upregulated in CSA patients. We tested the hypothesis that p122RhoGAP/DLC-1 overexpression causes coronary spasm.

**Methods and Results:**

We generated transgenic (TG) mice with vascular smooth muscle (VSM)-specific overexpression of p122RhoGAP/DLC-1. The gene and protein expressions of p122RhoGAP/DLC-1 were markedly increased in the aorta of homozygous TG mice. Stronger staining with anti-p122RhoGAP/DLC-1 in the coronary artery was found in TG than in WT mice. PLC activities in the plasma membrane fraction and the whole cell were enhanced by 1.43 and 2.38 times, respectively, in cultured aortic vascular smooth muscle cells from homozygous TG compared with those from WT mice. Immediately after ergometrine injection, ST-segment elevation was observed in 1 of 7 WT (14%), 6 of 7 heterozygous TG (84%), and 7 of 7 homozygous TG mice (100%) (p<0.05, WT versus TGs). In the isolated Langendorff hearts, coronary perfusion pressure was increased after ergometrine in TG, but not in WT mice, despite of the similar response to prostaglandin F_2α_ between TG and WT mice (n = 5). Focal narrowing of the coronary artery after ergometrine was documented only in TG mice.

**Conclusions:**

VSM-specific overexpression of p122RhoGAP/DLC-1 enhanced coronary vasomotility after ergometrine injection in mice, which is relevant to human CSA.

## Introduction

Coronary artery spasm plays an important role in the pathogenesis of Prinzmetal variant angina [1,2], myocardial infarction with nonobstructive coronary arteries [3], malignant ventricular arrhythmias [4,5], and the other acute coronary syndromes [6,7], all of which can cause sudden death. We and other investigators have shown that the basal vasomotor tone and constrictive response to diverse stimuli on the coronary artery are enhanced in Japanese patients with variant angina [7–10]. Importantly, all these stimuli exert their effects through receptors on plasma membrane and sequential cellular signaling mechanisms [11–15]. These findings suggest that intracellular and/or postreceptorial mechanisms are responsible for hyperactivity of vascular smooth muscle (VSM) [16]. The enhanced esophageal motility seen in patients with coronary spastic angina (CSA) further supports the idea that a generalized hyperactive VSM contraction is present in patients with CSA [17].

Phospholipase C (PLC), a key molecule for intracellular calcium regulation, produces inositol 1,4,5-trisphosphate (IP_3_) and diacylglycerol by hydrolyzing phosphatidylinositol 4,5-bisphosphate (PIP_2_). IP_3_ mobilizes Ca^2+^ from the intracellular stores and elicits rapid contraction of the VSM [18], whereas diacylglycerol activates protein kinase C and initiates sustained contraction by a Ca^2+^-independent mechanism [19]. We previously demonstrated that PLC activity in cultured skin fibroblasts obtained from patients with CSA was enhanced and a major PLC isozyme detected in the membrane fraction was the δ1 isoform which is known to be more sensitive to Ca^2+^ than the other isozymes [20].

A p122Rho GTPase-activating protein (GAP)/deleted in liver cancer-1 (p122RhoGAP/DLC-1) was recently cloned by screening a rat brain expression library with antiserum against purified PLC-δ1 and was identified as a dual functional molecule consisting of 1083 amino acid residues [21,22]. One function is an interaction with PLC-δ1 and enhancement of its activity to hydrolyze PIP_2_. The other one is a GAP activity specific for Rho [23]. A p122RhoGAP/DLC-1 is also recognized as a tumor suppressor, which is frequently down-regulated in several malignant cancers, such as colorectal, breast, prostate, and liver cancers [24]. However, its role in vascular system remains to be elucidated. We previously demonstrated that protein expression of p122RhoGAP/DLC-1 in cultured skin fibroblasts obtained from the CSA patients was upregulated by 3 times compared with control, and overexpression of p122RhoGAP/DLC-1 increased intracellular calcium concentration ([Ca^2+^]_i_) in response to acetylcholine [25]. However, it is unclear whether upregulation of p122RhoGAP/DLC-1 is a cause or result of coronary spasm. In the present study, we tested the hypothesis that VSM-specific p122RhoGAP/DLC-1 overexpression enhances PLCδ-1 activity and causes coronary spasm, which is relevant to human CSA.

## Methods

### Generation of transgenic (TG) mice with VSM-specific p122RhoGAP/DLC-1 overexpression

**Fig 1:**
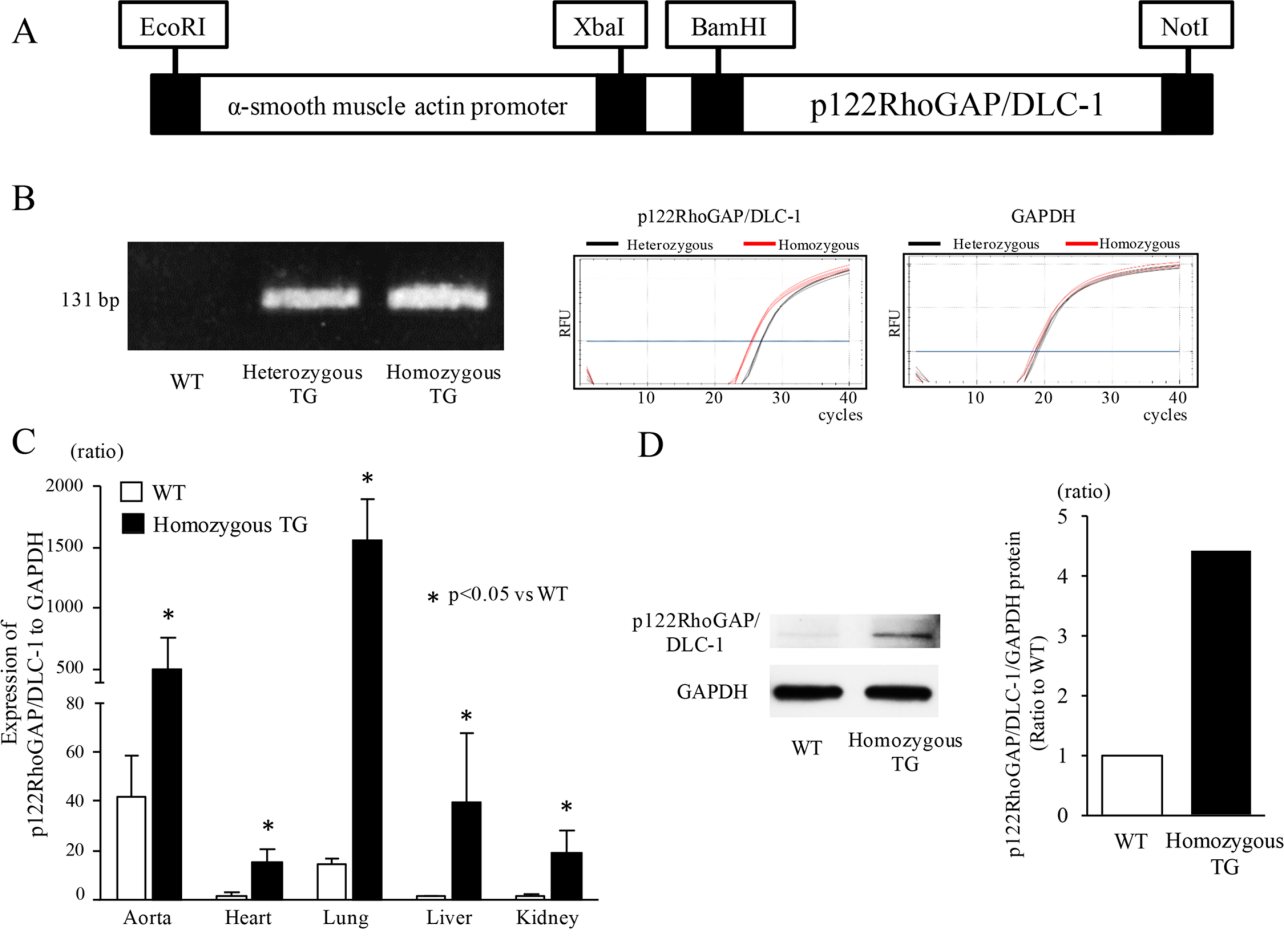
Generation of transgenic (TG) mice and their expression analyses. (A) Schematic map of the microinjected transgene consisting of the α-smooth muscle actin promoter and the mouse p122RhoGAP/DLC-1 cDNA. (B) Representative bands (131 bp) after genomic PCR and representative amplification curves for p122RhoGAP/DLC-1 and GAPDH in real-time PCR (40 cycles). (C) The gene expression of mouse p122RhoGAP/DLC-1 in various tissues of wild type (WT) (n = 3) and homozygous TG mice (n = 3). The ratio of p122RhoGAP/DLC-1 to GAPDH expression in the liver of WT mice was used as a reference (= 1), since it showed the lowest value among the tissues of WT mice. (D) Representative bands for mouse p122RhoGAP/DLC-1 protein in the pooled aortas (left panel) and densitometric analysis between WT and homozygous TG mice (right panel). The aortas from 4 mice in WT and TG.

The mouse p122RhoGAP/DLC-1 cDNA was subcloned into plasmid pBsKS(-) including 4.7 kb fragment of the mouse α-smooth muscle actin (SMA) promoter. The resultant recombinant construct was digested with EcoRI and NotI to generate a 7.3-kb DNA fragment consisting of the α-SMA promoter and the mouse p122RhoGAP/DLC-1 cDNA (Fig 1A). The DNA fragment was then microinjected into the pronuclei of fertilized mouse embryos at the single-cell stage to generate TG mice (C57BL/6J strain), as reported previously [26–28]. Two lines (line 1, line 2) of homozygous TG mice were finally generated. Because they had similar phenotypes and responses to stimuli, the data on line1 of TG mice have been shown in the following experiments. All animals were maintained in the same environment, including constant temperature and humidity, and free access to food and water. The experiments were conducted in TG and WT mice at the age of 20 through 30 weeks unless otherwise noted. All procedures were performed in accordance with the Guide for the Care and Use of Laboratory Animals of the National Institutes of Health (NIH) and were reviewed and approved by the Institutional Animal Care and Use Committee of Hirosaki University Graduate School of Medicine (Permit number: G15002). Mice were euthanized by cervical dislocation under anesthesia with medetomidine (0.75 mg/kg), midazolam (4 mg/kg), and butorphanol tartrate (5 mg/kg) by intraperitoneal route for the following experiments.

### Genotyping and gene expression analyses

Genomic DNA was extracted from mice toe or finger and genotyping was performed by PCR using the following transgene-specific primers: 5'-AGGATAGCACTAGGACACAGAGGA-3' for sense primer and 5'- GCTGTACGAAGATCTACTGTTCCC-3' for anti-sense primer. Heterozygous and homozygous TG mice were determined by semiquantitative analysis of genomic DNA using TaqMan Universal PCR Master Mix (Applied Biosystems, Foster City, CA) as a relative ratio of transgene to GAPDH. The sense and anti-sense primers for the transgene were 5'-TTACAGCTGAGGCTGCTTC-3' and 5'-CAAGCTTCCTTGGCTTCAAT-3', respectively. The TaqMan probe was 5-GTGTAGAGACGAGCCGGACACC-3'.

After the euthanasia of mice, the tissues were immediately excised. Total RNA was extracted from homogenized descending aorta, heart, lung, liver, and kidney with the use of RNeasy Protect Mini Kit or RNeasy Fibrous Tissue Mini Kit according to the protocol of the manufacturer (Qiagen, Valencia, CA). Reverse transcription was performed by using PrimeScript™ 1st strand cDNA Synthesis Kit (Takara Bio, Shiga, Japan). For semiquantitative analysis of gene expression, TaqMan Universal PCR Master Mix (Applied Biosystems, Foster City, CA) and specific primers for mouse p122RhoGAP/DLC-1 and GAPDH were used. The sense and anti-sense primers for p122RhoGAP/DLC-1 were 5'-GACACCATGATCCTAACAC-3' and 5'- CAACAGGGAACAGTAGATC-3', respectively. The TaqMan probe was 5'-GGAAGCTTGCGACTGGCTGA-3'.

### Weight measurement of the lung and heart, and Fulton index calculation

After anesthetized with medetomidine (0.75 mg/kg), midazolam (4 mg/kg) and butorphanol tartrate (5 mg/kg) by intraperitoneal route, mice were sacrificed for tissue isolation by cervical dislocation. The right ventricle (RV) was dissected from the left ventricle plus septum (LV+S). We measured the weights of the lung, heart, LV+S, and RV, and then Fulton index was calculated as RV/ (LV+S) to evaluate RV hypertrophy [29] [30].

### Western blot analysis

After the euthanasia of mice, the aortas were immediately excised. The aortas from 4 mice were pooled, homogenized, and centrifuged in RIPA lysis buffer system (Santa Cruz Biotechnology, Santa Cruz, CA), and the supernatant was collected for western blot analysis. Protein concentrations were determined using bicinchoninic acid (BCA) Protein Assay (Pierce, Rockford, IL, USA). We loaded protein samples containing 30 μg protein per analyzed band. The protein was separated by sodium dodecyl sulfide-polyacrylamide gel electrophoresis and electrophoretically transferred to a polyvinylidene fluoride membrane (Bio-Rad Laboratories). After blocking for 1 hour, the membranes were incubated with the primary antibody for p122RhoGAP/DLC-1 (sc-32931; Santa Cruz Biotechnology) and GAPDH (sc-25778; Santa Cruz Biotechnology), both diluted 1:1000 at 4°C overnight. Horseradish peroxidase and alkaline phosphatase conjugated anti-rabbit antibody (sc-2004; Santa Cruz Biotechnology) diluted 1:5000 was used as a secondary antibody. The protein bands were detected by Amersham ECL Prime Western Blotting Detection Reagents (GE Healthcare, Buckinghamshire, UK). Densitometric analysis was performed with Scion image software, and a relative ratio of the target protein to GAPDH was calculated in each sample.

### Immunofluorescence microscopy

After the euthanasia of mice, the heart was excised, frozen in liquid nitrogen-cooled isopentane, sectioned from base to apex at a thickness of 6 μm, and then fixed in -20°C methanol for 10 minutes. The tissue sections were treated with the primary antibody for p122RhoGAP/DLC-1 (sc-28434; Santa Cruz Biotechnology) and α-SMA (ab5694; Abcam, Cambridge, UK), both were diluted 1:1000 for 12 hours at 4°C. After washing repeatedly in PBS, the sections were covered with PBS containing secondary antibody conjugated with Alexa Fluor 488 (A11055; Thermo Fisher Scientific, Waltham, MA) and Alexa Fluor 568 (A10042; Thermo Fisher Scientific), both were diluted 1:1000 for 1 hour at room temperature and then rinsed with PBS. Fluorescent images were captured with the use of fluorescence microscope (BZ-X700; Keyence, Osaka, Japan). To differentiate nonspecific binding of antibodies, isotype-matched control normal rabbit IgG (sc-2027; Santa Cruz Biotechnology) or normal goat IgG (sc-2028; Santa Cruz Biotechnology) was applied and incubated under the same conditions.

### Blood pressure measurement

Blood pressure was measured by tail-cuff method (BP-98A; Softron, Tokyo, Japan) in conscious mice, which were acclimated to restraint and tail-cuff inflation for one week prior to the measurement. After the highest and lowest readings were discarded, at least 10 readings were averaged each day for continuous 3 days.

### Echocardiography

Transthoracic M-mode image obtained from the short-axis view of the left ventricle (LV) with the use of a Philips HD11 XE and a 15 MHz linear probe were recorded in mice anesthetized with medetomidine (0.75 mg/kg), midazolam (4 mg/kg) and butorphanol tartrate (5 mg/kg) by intraperitoneal route. LV end-systolic dimension (LVESD), LV end systolic dimension (LVEDD), and LV posterior wall thickness were measured, and the data from at least 3 cardiac cycles were averaged. LV fractional shortening (%) was calculated as [(LVEDD-LVESD)/LVEDD] × 100.

### PLC activity in cultured vascular smooth muscle cells

After the euthanasia of mice, the ascending and descending aortas were immediately dissected from the TG and WT mice. Primary aortic vascular smooth muscle cells (VSMCs) were cultured by the explant method as described previously [31]. VSMCs were cultured in 5% CO_2_ in air at 37°C in Dulbecco’s modified Eagle’s medium (DMEM) (# 11965–092, Gibco) supplemented with 10% Fetal Bovine Serum (#12483–020, Gibco), penicillin (100 U/ml) and streptomycin (100 μg/ml) (#15140–122, Gibco). VSMCs were subcultured using trypsin/EDTA when they reached about 80% confluence. For experiments, VSMCs at passage 2 to 4 were used. The differentiated phenotype of VSMCs was verified by immunostaining using antibodies against α-SMA (ab5694; Abcam, Cambridge, UK) and SM22α (ab14106; Abcam, Cambridge, UK).

Confluent monolayers were scraped and homogenized as described previously [32]. The homogenate was centrifuged at 500 g for 10 min, and the supernatant was centrifuged at 40,000 g for 15 min. The pellet and supernatant were stored as a plasma membrane fraction and as a cytoplasmic fraction, respectively, at -80°C. The protein content was measured spectrophotometrically. We measured PLC activities using the plasma membrane fraction of cultured VSMCs and the whole cells including the plasma membrane and the cytoplasmic fractions. The PLC assay system consists of the following components: N-2- hydroxyethylpiperazine-N’-2-ethanesulfonic acid (50 mmol/L), CaCl_2_ (0.1 mmol/L), sodium cholate (9 mmol/L), ^3^H-PIP_2_ (40,000 cpm), and the cell protein (20 μg). The reaction was stopped with chloroform/methanol/HCl followed by 1N HCl containing EGTA. After extraction, the aqueous phase was removed for liquid scintillation counting.

### ECG recordings and response to ergometrine

Ergometrine maleate (30 mg/mL in saline) at 50 mg/kg (Sigma, St Louis, MO), which stimulates the serotonergic receptor directly and triggers VSM constriction [33], was injected into the jugular vein of the anesthetized mice over 10 minutes, as previously described [34]. The ECG lead II was recorded continuously before and after ergometrine injection and was analyzed by the investigators blinded to mouse genotype.

### Coronary perfusion pressure in isolated Langendorff-perfused hearts

The TG and WT mice were heparinized (0.5 U/g) and anesthetized with an intraperitoneal injection of a mixture of medetomidine (0.75 mg/kg), midazolam (4 mg/kg) and butorphanol tartrate (5 mg/kg). The hearts were then rapidly excised and transfused via a 19-gauge cannula (Psysio-tech, Tokyo, Japan) that was placed immediately distal of the intact aortic valve, as described previously [34,35]. The hearts were perfused at a constant flow (1.25 mL/min) with Krebs-Henseleit solution (in mmol/L:120 NaCl, 4.7 KCl, 1.2 MgSO_4_, 1.2 KH_2_PO_4_, 10 glucose, 25 NaHCO_3_, 1.25 CaCl_2_) equilibrated with 95% O_2_ and 5% CO_2_ at 37°C with the use of a standard Langendorff setup (Psysio-tech). Coronary perfusion pressure was recorded continuously with the use of a pressure-sensing catheter (AD Instruments, Bella Vista, Australia) connected to the perfusion cannula. The hearts were equilibrated for at least 20 minutes before experiments. Each of ergometrine and prostaglandin F_2α_ (PGF_2α_) was given in the perfusion solution at the final concentration of 1 and 10 μmol/L, respectively, in Langendorff-perfused hearts for 20 minutes. During the experiment, the hearts were maintained at 38°C via a water-jacketed tissue-organ bath.

### Microvascular filling

The microvascular filling experiment was performed as previously described [34,36]. The TG and WT mice were anesthetized with an intraperitoneal injection of a mixture of medetomidine, midazolam, and butorphanol tartrate, and then the hearts were rapidly excised and transfused via a 19-gauge cannula that was placed immediately distal of the intact aortic valve. The hearts were then perfused at a constant flow (1.25 mL/min) with Krebs-Henseleit solution equilibrated with 95% O_2_ and 5% CO_2_ at 37°C with the use of a standard Langendorff setup. Coronary arteries were perfused by either ergometrine (1 μmol/L) for 20 minutes or vehicle and followed by the infusion of Microfil, a liquid latex medium (Flow Tech, Inc. Carver, MA). The images of the coronary arteries filled with the Microfil were captured by using X-Ray Inspection Systems (YXLON International K.K., Kanagawa, Japan).

### Statistical analysis

All continuous data are expressed as mean±SD. One-way ANOVA followed by Bonferroni multiple comparison tests was used for the analyses of ECG and coronary perfusion pressure studies. Differences in proportions were analyzed by χ2 test or Fisher exact probability test. P<0.05 was considered as a statistical significance.

## Results

### VSM-specific overexpression of p122RhoGAP/DLC-1 in TG mice

The mouse p122RhoGAP/DLC-1 transgene was detected only in TG mice by genomic PCR and the distinction between homozygous and heterozygous TG mice was confirmed by real-time PCR (Fig 1B), where red line showing homozygous p122RhoGAP/DLC-1 TG mouse shifted to the left by approximately one cycle. The gene expression levels of total p122RhoGAP/DLC-1 (endogenous plus transgene) in various tissues including the aorta, heart, liver, lung, and kidney are shown in Fig 1C. The ratio of p122RhoGAP/DLC-1 to GAPDH expression in WT mice was the highest in the aorta, followed by the lung, kidney, heart, and liver. Markedly higher expression levels of p122RhoGAP/DLC-1 were found in homozygous TG mice compared with WT mice, especially in the lung and aorta. The protein expression of p122RhoGAP/DLC-1 in the aorta was also increased by 4.4 times in homozygous TG compared with WT mice (Fig 1D).

**Fig 2:**
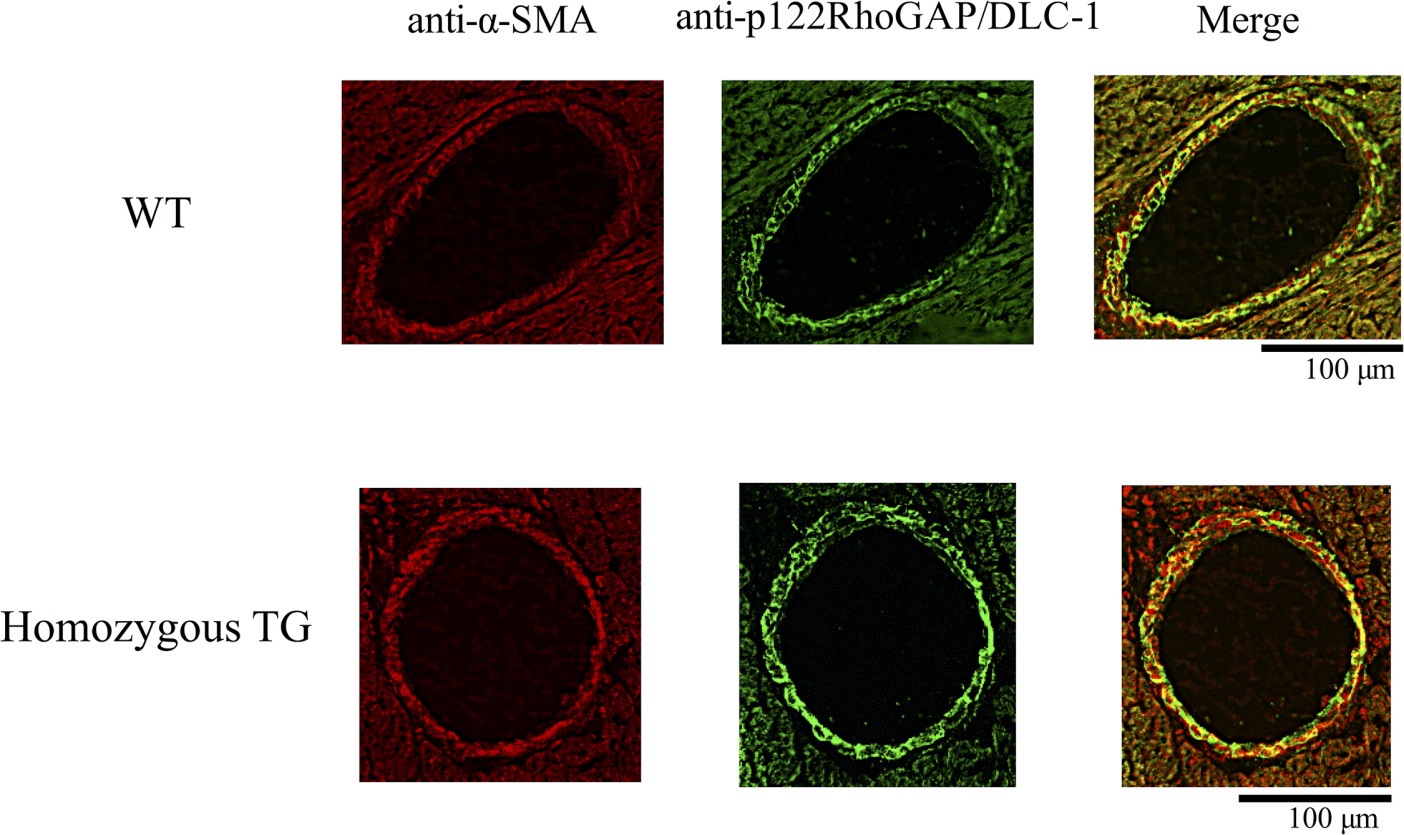
Immunofluorescence images of the coronary artery. Representative images from 3 WT and 3 TG mice are shown. Left panels show anti-α-smooth muscle actin (SMA) reactivity to mark vascular smooth muscle cells (red). Middle panels show staining with an anti-p122RhoGAP/DLC-1 antibody demonstrating the increase in p122RhoGAP/DLC-1 immunoreactivity in homozygous TG mice (green). Right panels show merged images.

Representative immunofluorescence images of heart section from WT and TG mice are shown in Fig 2. The whole media of large coronary arteries was stained by α-SMA (left panel) and p122RhoGAP/DLC-1 (middle panel) in both WT and homozygous TG mice. However, more strongly stained whole media with anti-p122RhoGAP/DLC-1 was found in homozygous TG mice than in WT mice, indicating that mouse p122RhoGAP/DLC-1 was overexpressed in TG mice. The merged images of anti-p122RhoGAP/DLC-1 and anti-α-SMA antibodies (right panel) confirmed that p122RhoGAP/DLC-1 was present in VSM of the coronary arteries.

### Basic phenotypes of TG mice

Neonatal mortality was not significantly different between TG and WT mice. Neither homozygous nor heterozygous TG mice showed early mortality. Systolic blood pressure was slightly but significantly elevated in homozygous TG mice (n = 28) compared with WT mice (n = 24) at the age of 12–18 weeks (99±9 vs 92±8 mm Hg, p<0.05). Echocardiographic study demonstrated that LV fractional shortening, LVESD, LVEDD, and LV posterior wall thickness were not statistically different between homozygous TG and WT mice at the age of 20 weeks (Table 1).

**Table 1:** Echocardiographic analyses in wild type (WT) and homozygous p122RhoGAP/DLC-1 TG mice.

	WT mice (n = 4)	Homozygous TG mice (n = 4)
Interventricular septum, mm	0.88 ± 0.06	0.83 ± 0.03
LV posterior wall, mm	0.83 ± 0.09	0.80 ± 0.04
LV end-diastolic dimension, mm	3.76 ± 0.10	3.71 ± 0.17
LV fractional shortening, %	38.5 ± 0.45	38.5 ± 0.28

Data are shown as mean±SD. LV indicates left ventricular.

To investigate the pulmonary congestion and RV hypertrophy, we measured weights of the lung, heart, LV+S, and RV, and then calculated the Fulton index. As shown in S1 Table, there were no significant differences in the weights of the lung and heart, and Fulton index between TG and WT mice.

### PLC activity in cultured VSMCs

**Fig 3:**
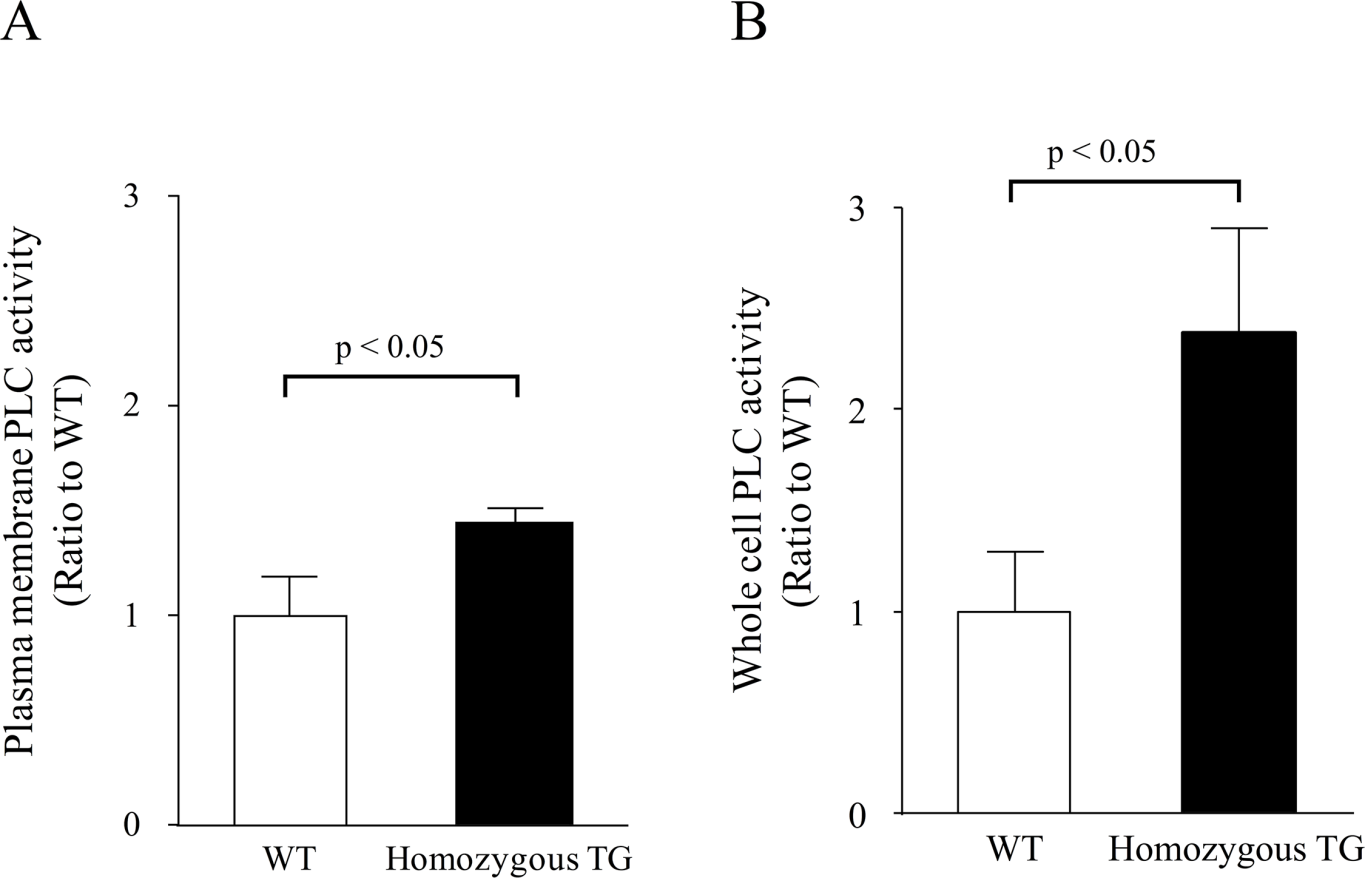
PLC activity in cultured vascular smooth muscle cells. (A) PLC activity in the plasma membrane fraction (n = 3 in WT and TG mice). (B) PLC activity in the whole cell (n = 3 in WT and TG mice).

As shown in Fig 3, PLC enzymatic activity in the plasma membrane fraction of cultured aortic VSMCs was increased by 1.43±0.07 times in homozygous TG mice compared with WT mice (both n = 3, p<0.05). PLC activity in the whole cell was also significantly higher (2.38±0.51 times) in homozygous TG mice than in WT mice (both n = 3, p<0.05).

### ECG changes in TG mice after ergometrine injection

**Fig 4:**
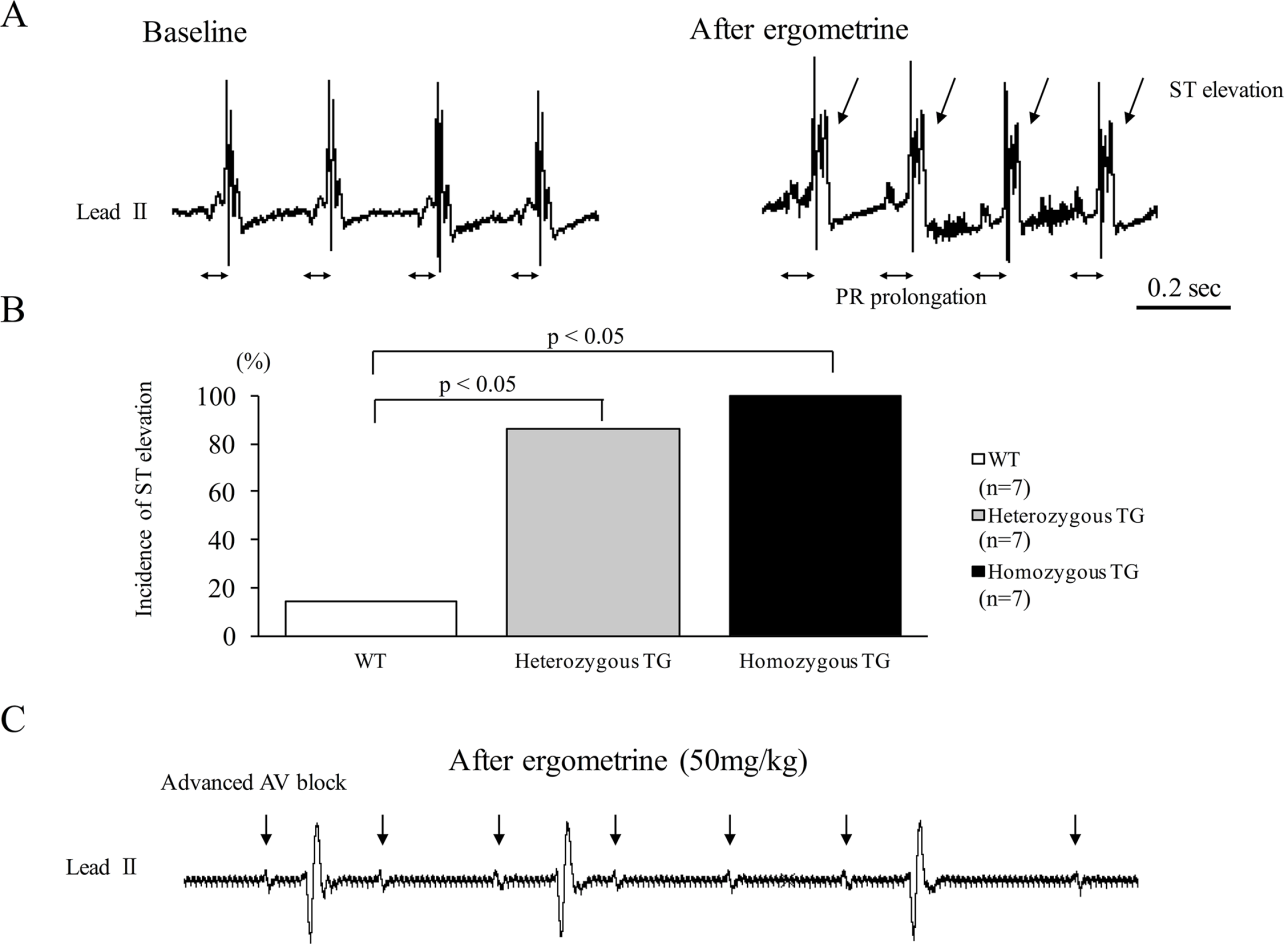
Representative ECG recordings and responses to ergometrine in wild type (WT) and transgenic (TG) mice. **(**A) Representative ECG recordings before (left) and after (right) intravenous injection of ergometrine in anesthetized homozygous TG mouse. Ergometrine injection immediately elicited ST-segment elevation with PR prolongation. (B) Incidence of ST-segment elevation after ergometrine injection in WT, heterozygous, and homozygous TG mice. (C) Representative ECG showing advanced AV block in homozygous TG mice after ergometrine injection.

There were no differences in heart rate, QRS duration, and PR interval at baseline among WT, heterozygous, and homozygous TG mice (S2 Table). As shown in Fig 4A, intravenous injection of ergometrine at 50 mg/kg to the anesthetized homozygous TG mice promptly induced ST-segment elevation (indicated by arrows) associated with PR-interval prolongation (indicated by bars). ST elevation caused by ergometrine at 50 mg/kg was observed in 1 of 7 WT (14%), 6 of 7 heterozygous TG (84%), and 7 of 7 homozygous TG mice (100%) (p<0.05, WT versus TGs) (Fig 4B). ST elevation was also observed in homozygous TG of another line (line2, data not shown). As shown in Fig 4C, ST-segment elevation after ergometrine was sometimes followed by complete or advanced atrioventricular block.

### Coronary perfusion pressure in isolated Langendorff-perfused heart

**Fig 5:**
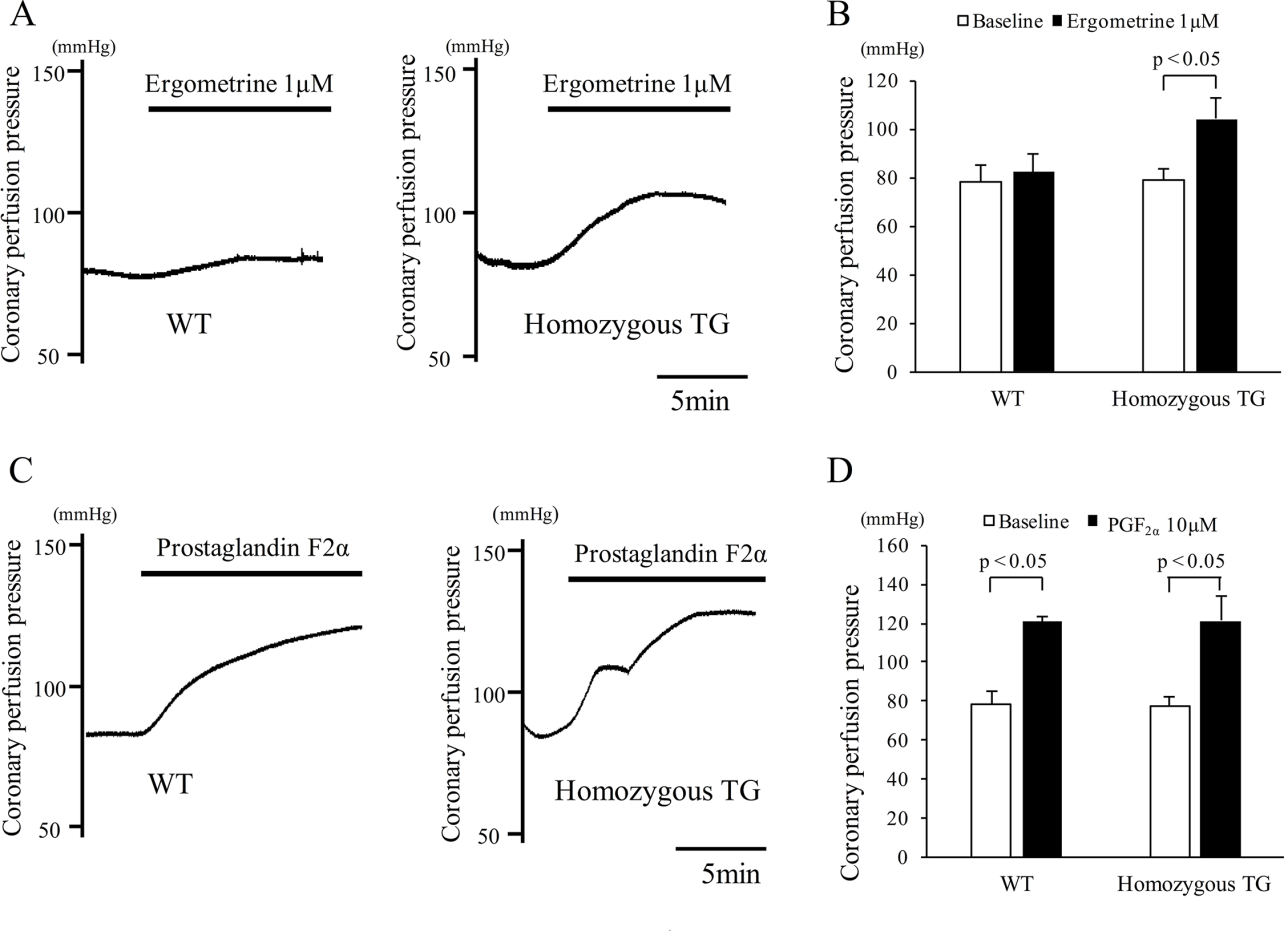
Coronary perfusion pressures in isolated Langendorff hearts of wild type (WT) and homozygous transgenic (TG) mice. (A) Representative traces of coronary perfusion pressures before and after injection of ergometrine at 1 μmol/L in WT (left) and homozygous TG (right) mice. (B) Changes in coronary perfusion pressure before (open bar) and after (closed bar) injection of ergometrine at 1 μmol/L in WT (n = 5) and homozygous TG mice (n = 5). (C) Representative traces of coronary perfusion pressures before and after injection of prostaglandin F_2α_ (PGF_2α_) at 10 μmol/L in WT (left) and homozygous TG (right) mice. (D) Changes in coronary perfusion pressure before (open bar) and after (closed bar) injection of PGF_2α_ at 10 μmol/L in WT (n = 3) and homozygous TG mice (n = 3).

Representative coronary perfusion pressures after treatment with ergometrine at 1 μmol/L are shown in Fig 5A. Coronary perfusion pressure at baseline was similar between WT and homozygous TG mice, whereas it was significantly increased in homozygous TG mice by ergometrine administration, but not in WT mice (both n = 5, p<0.05) (Fig 5B). Coronary perfusion pressure was increased after PGF_2α_ at 10 μmol/L to a similar degree in both mice (Fig 5C), and no statistical difference was found between WT and homozygous TG mice (both n = 3) (Fig 5D).

### Microvascular filling experiment

**Fig 6:**
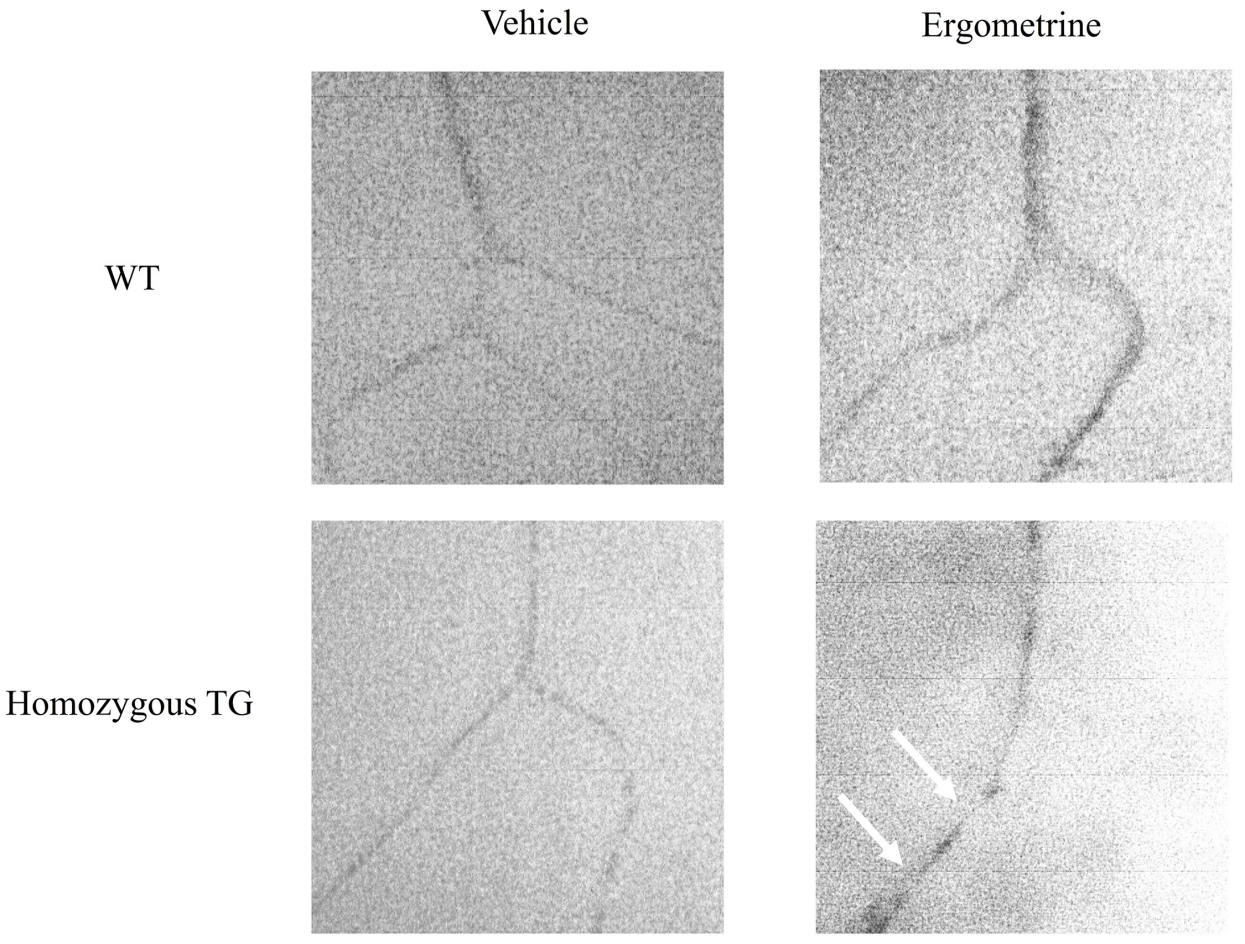
Microvascular filling experiment with microfil of the coronary arteries in wild type (WT) and homozygous transgenic (TG) mice. In the group treated with vehicle, no focal narrowing was observed in either WT (n = 3) or TG mice (n = 3). Focal narrowing (arrows) of the coronary artery after injection of ergometrine was observed in homozygous TG (3 of 3), but not in WT mice (0 of 3).

Fig 6 shows representative images of the coronary arteries of WT and homozygous TG mice treated with vehicle or ergometrine at 1 μmol/L. In the group treated with vehicle, no focal spasm was observed in any portions of the artery in either WT or homozygous TG mice (both n = 3). In the group treated with ergometrine, focal narrowing of the coronary artery was documented in homozygous TG mice (3 of 3), but not in WT mice (0 of 3).

## Discussion

### Major findings

In the present study, we generated TG mice with VSM-specific overexpression of p122RhoGAP/DLC-1 and investigated whether this newly developed TG mice cause a coronary spasm. Histological and biochemical analyses showed an increased expression of p122RhoGAP/DLC-1 in the coronary artery wall and an enhanced PLC activity in cultured aortic VSMCs of TG mice. Functional analyses on the vascular tone of the coronary arteries in TG mice further showed that ergometrine administration caused ST-segment elevation on ECG, focal narrowing of the coronary artery by the microvascular filling experiment, and an elevated coronary perfusion pressure by the Langendorff-perfused experiment. All these findings strongly indicate that upregulation of p122RhoGAP/DLC-1 in the coronary arteries plays a causal role in the pathogenesis of coronary spasm, which is relevant to human CSA.

### Demonstration of coronary spasm induced by ergometrine in mice with VSM-specific overexpression of p122RhoGAP/DLC-1

We previously showed that the mutation of PLC-δ1 at 864 G to A, accompanied by amino acid replacement of arginine 257 to histidine (R257H), are present in CSA patients, and significantly enhances PLC enzymatic activity in the physiological range of [Ca^2+^]_i_ [37]. To further investigate its role in coronary spasm, we generated mice overexpressing variant PLC-δ1 (R257H) under control of the mouse α-SMA promoter, and demonstrated that increased PLC-δ1 activity causes enhanced coronary vasomotility such as that seen in patients with CSA [34]. On the other hand, we recently found elevated gene and protein expressions of p122RhoGAP/DLC-1 in the cultured skin fibroblasts obtained from CSA patients [25]. Notably, the ratio of protein expression of p122RhoGAP/DLC-1 to GAPDH protein in CSA patients ranged from 1.5 to 3.9 and all of them were greater than the value of plus 1 sigma (0.4) of the mean value in the control subjects. To clarify the etiologic role of enhanced p122RhoGAP/DLC-1 in coronary spasm, we generated mice with VSM-specific overexpression of p122RhoGAP/DLC-1 in the present study. Successful generation of TG mice was confirmed by showing that there was a strong immunofluorescence signal for p122RhoGAP/DLC-1 merged with α-SMA in the coronary arteries of TG mice, and that the protein expression of p122RhoGAP/DLC-1 in the aorta of TG mice was increased by approximately 4-fold compared with WT mice.

Coronary vasomotility in this newly developed TG mice was examined. Ergometrine is an ergot alkaloid that stimulates serotonergic receptors and triggers contraction of the VSMCs [35]. This contraction is dependent on Ca^2+^ mobilization and not on Ca^2+^ sensitization. We examined coronary vasomotility in response to ergometrine. First, ECG changes after intravenous injection of ergometrine were studied. Previous reports showed that a spontaneous ST-segment elevation followed by atrioventricular block was observed in SUR-null and Kir6.1-null mice [35,38,39]. We also previously showed a ST-segment elevation followed by atrioventricular block after intravenous injection of ergometrine in variant PLC-δ1 TG mice [34]. In the present study, a spontaneous ST-segment elevation did not occur, but ergometrine injection induced ST-segment elevation in all homozygous TG mice, followed by atrioventricular block in some TG mice. Second, changes in coronary perfusion pressure after ergometrine administration were evaluated by isolated Langendorff perfused hearts. A previous study in variant PLC-δ1 TG mice revealed an increase in the coronary perfusion pressure after ergometrine administration [34]. The present study also demonstrated that the coronary perfusion pressure after ergometrine administration was increased only in homozygous TG mice, but not in WT mice. Third, coronary artery was visualized by microvascular filling technique. Spontaneous focal coronary artery narrowing was shown in SUR-null mice with the use of this technique [36]. In the present study, focal coronary artery narrowings after ergometrine administration were observed only in homozygous TG mice, but not in WT mice. All these findings of the present study support our hypothesis that upregulation of p122RhoGAP/DLC-1 in the coronary artery smooth muscles induces coronary spasm and may play a causal role in the pathogenesis of human CSA.

### Possible mechanisms of coronary spasm induced by VSM-specific overexpression of p122RhoGAP/DLC-1

The p122RhoGAP/DLC-1 has an interaction with PLC-δ1 and enhances its activity to hydrolyze PIP_2_ [23]. In the present study, we measured PLC activity in the plasma membrane fraction and in the whole cell using cultured aortic VSMCs obtained from TG and WT mice. We found that PLC activity in the plasma membrane fraction was increased by 1.43 times in TG mice compared with WT mice. Furthermore, its activity in the whole cell was 2.38 times higher in TG mice than in WT mice. The disparity of PLC activity between the membrane fraction and the whole cell is likely to be associated with the subcellular localization of p122RhoGAP/DLC-1 and PLC-δ1. We previously reported that PLC activity in the cultured skin fibroblasts obtained from patients with CSA was enhanced by 2.7 times compared with that from control subjects without angina pectoris [20]. Thus, the increased PLC activity found in p122RhoGAP/DLC-1 TG mice is likely responsible for enhanced coronary vasomotility and is a possible mechanism for coronary spasm.

Yamaga et al studied the interaction between p122RhoGAP/DLC-1 and PLC-δ1 in more details using rat pheochromocytoma PC12 cells [40]. The agonist-induced increase in [Ca^2+^]_i_ recruits PLC-δ1 from the cytoplasm into lipid rafts, where PLC-δ1 interacts with p122RhoGAP/DLC-1. This complex, in turn, leads to a robust hydrolysis of PIP_2_ and subsequent increase in [Ca^2+^]_i_ via IP_3_ receptor activation. Furthermore, the increased [Ca^2+^]_i_ stimulates activation of store-operated channels such as transient receptor potential channels to increase Ca^2+^ influx with a positive feedback mechanism. However, there have been no reports regarding the role of p122RhoGAP/DLC-1 in VSMCs. By using HEK 293 and A7r5 aortic smooth muscle cells, we previously demonstrated that [Ca^2+^]_i_ at baseline and the peak increase in [Ca^2+^]_i_ in response to acetylcholine were both significantly higher in cells transfected with p122RhoGAP/DLC-1 than in those without p122RhoGAP/DLC-1 [25]. Conversely, knockdown of p122RhoGAP/DLC-1 resulted in diminished [Ca^2+^]_i_ response in human coronary artery smooth muscle cells [25]. Taken together, similar interactions among p122RhoGAP/DLC-1, PLC-δ1, and Ca^2+^ signaling as seen in rat pheochromocytoma PC12 cells might be present in the coronary artery smooth muscle cells of the p122RhoGAP/DLC-1 TG mice. However, more studies in this regard are needed.

The p122RhoGAP/DLC-1 not only functions as a PLC-δ1 activator but also has a GAP activity, which inactivates small G-protein Rho. In the present study, only homozygous TG mice showed the elevated coronary perfusion pressure after ergometrine administration in isolated Langendorff perfused hearts, whereas both TG and WT mice showed the elevated coronary perfusion pressure after PGF_2α_ administration. It is known that PGF_2α_ elicits vasoconstriction by an actin-associated mechanism for RhoA kinase activation [41]. These findings indicate that Rho-GAP cascade may not be involved in the enhanced coronary vasomotility in this TG mice.

Our model is transgenic mice, in which a number of transgene copies are randomly integrated into the genome. In this context, we cannot completely exclude the possibility that integration of the transgenes may disrupt the original genes. However, the finding that 2 lines of our TG mice showed ST-segment elevation by ergometrine may not support possible involvement of the gene disruption in the present study.

As expression levels of p122RhoGAP/DLC-1 were the highest in the lung, our mice may have some phenotypes such as pulmonary hypertension due to enhanced pulmonary arterial vasomotility. Therefore, we measured weights of the lung, heart, LV+S, and RV, and then Fulton index was calculated to evaluate RV hypertrophy. The results showed that there were no significant differences in the weights of the lung and heart, and Fulton index between TG and WT mice (S1 Table). These findings indicate that obvious pulmonary congestion or RV hypertrophy has not occurred in our TG mice.

### Clinical implications of our findings

Several experimental animal models for coronary spasm were reported. In the Kir6.1-null or SUR-null mouse, spontaneous coronary spasm occurred and consequently led to sudden death [38,39]. However, the relevance of these animal models in the clinical setting remains unclear. We and others showed that no mutation that alters primary structure of Kir6.1 was detected in 19 Japanese [42] or 18 Italian patients with CSA [43]. Furthermore, we found no genetic mutation associated with amino acid substitution of SUR in 9 Japanese CSA patients [44]. These results indicate that abnormality in the primary structure of SUR and Kir6.1 may not be involved in the genetic pathogenesis of CSA in humans.

We previously showed that the 864 G to A mutation of PLC-δ1 was present only in 10% of male CSA patients [37]. This suggests the presence of other factors involved in the pathogenesis of coronary spasm. Thus, the present study raised a possibility that enhanced p122RhoGAP/DLC-1 expression may be a major cause of CSA [25]. Our TG mice generated in the present study may help further understand the pathogenesis of CSA, as well as the variant PLC-δ1 (R257H) TG mice.

## Conclusions

VSM-specific overexpression of p122RhoGAP/DLC-1 enhanced coronary vasomotility after ergometrine injection in mice, which is relevant to human CSA. Further clinical investigation is required to clarify clinical and pathological importance of p122RhoGAP/DLC-1 in patients with CSA.

## Supporting Information

S1 Table Weights of the body, lung, and heart, and Fulton index of wild type (WT) and homozygous p122RhoGAP/DLC-1 TG mice. (DOC)

S2 Table HR, QRS duration, and PR interval at baseline in wild type (WT), heterozygous, and homozygous p122RhoGAP/DLC-1 TG mice. (DOC)
