# Supplementary material for: Enhanced p122RhoGAP/DLC-1 Expression Can Be a Cause of Coronary Spasm
Source: PLoS One. 2015 Dec 1;10(12):e0143884. doi: 10.1371/journal.pone.0143884 (PMC4666625; doi:10.1371/journal.pone.0143884)
Supplement: S1 Table — (DOC) [file pone.0143884.s001.doc]

**Supporting information Table 1.** Weights of the body, lung, and heart, and Fulton index of wild type (WT) and homozygous p122RhoGAP/DLC-1 TG mice

|  | WT | Homozygous TG | p value |
| --- | --- | --- | --- |
| BW | 31.7 ± 2.28 | 31.6 ± 2.09 | NS |
| Lung (Right) (mg/g) | 2.64 ± 0.10 | 2.63 ± 0.10 | NS |
| Lung (Left) (mg/g) | 2.22 ± 0.11 | 2.1 ± 0.11 | NS |
| LV+S (mg/g) | 3.21 ± 0.51 | 3.15 ± 0.16 | NS |
| RV (mg/g) | 0.76 ± 0.11 | 0.77 ± 0.06 | NS |
| Fulton index | 0.24 ± 0.01 | 0.25 ± 0.02 | NS |

BW = Body weight, LV+S = Left ventricle+septum, RV = Right ventricle.

Fulton index was calculated as RV/(LV+S)
