# Supplementary material for: Enhanced p122RhoGAP/DLC-1 Expression Can Be a Cause of Coronary Spasm
Source: PLoS One. 2015 Dec 1;10(12):e0143884. doi: 10.1371/journal.pone.0143884 (PMC4666625; doi:10.1371/journal.pone.0143884)
Supplement: S2 Table — (DOC) [file pone.0143884.s002.doc]

**Supporting information Table 2**. HR, QRS duration, and PR interval at baseline in wild type (WT), heterozygous, and homozygous p122RhoGAP/DLC-1 TG mice

|  | WT (n=7) | Heterozygous TG (n=7) | Homozygous TG (n=7) |
| --- | --- | --- | --- |
| HR (beats/min) | 280.1 ± 44.0 | 272.4 ± 42.8 | 259.3 ± 51.2 |
| QRS duration (ms) | 33.1 ± 6.0 | 31.4 ± 6.1 | 33.3 ± 4.1 |
| PR interval (ms) | 43.7 ± 7.2 | 43.1 ± 5.4 | 44.3 ± 5.0 |

HR = heart rate. There were no differences in HR, QRS duration, and PR interval at baseline among WT, heterozygous, and homozygous TG mice.
